# E-Selectin-Overexpressing Mesenchymal Stem Cell Therapy Confers Improved Reperfusion, Repair, and Regeneration in a Murine Critical Limb Ischemia Model

**DOI:** 10.3389/fcvm.2021.826687

**Published:** 2022-01-31

**Authors:** Hallie J. Quiroz, Samantha F. Valencia, Hongwei Shao, Yan Li, Yulexi Y. Ortiz, Punam P. Parikh, Roberta M. Lassance-Soares, Roberto I. Vazquez-Padron, Zhao-Jun Liu, Omaida C. Velazquez

**Affiliations:** ^1^Division of Vascular Surgery, DeWitt-Daughtry Family Department of Surgery, University of Miami Miller School of Medicine, Miami, FL, United States; ^2^Vascular Biology Institute, University of Miami Miller School of Medicine, Miami, FL, United States

**Keywords:** cell therapy, E-selectin, adeno-associated virus, limb salvage, angiogenesis

## Abstract

**Aims:**

Novel cell-based therapeutic angiogenic treatments for patients with critical limb ischemia may afford limb salvage. Mesenchymal stem cells (MSCs) do not overexpress E-selectin; however, we have previously demonstrated the cell-adhesion molecule's vital role in angiogenesis and wound healing. Thus, we created a viral vector to overexpress E-selectin on MSCs to increase their therapeutic profile.

**Methods and Results:**

Femoral artery ligation induced hind limb ischemia in mice and intramuscular injections were administered of vehicle or syngeneic donor MSCs, transduced *ex vivo* with an adeno-associated viral vector to express either GFP^+^ (MSC^GFP^) or E-selectin-GFP^+^ (MSC^E−selectin−GFP^). Laser Doppler Imaging demonstrated significantly restored reperfusion in MSC^E−selectin−GFP^-treated mice vs. controls. After 3 weeks, the ischemic limbs in mice treated with MSC^E−selectin−GFP^ had increased footpad blood vessel density, hematoxylin and eosin stain (H&E) ischemic calf muscle sections revealed mitigated muscular atrophy with restored muscle fiber size, and mice were able to run further before exhaustion. PCR array-based gene profiling analysis identified nine upregulated pro-angiogenic/pro-repair genes and downregulated *Tumor necrosis factor (TNF)* gene in MSC^E−selectin−GFP^-treated limb tissues, indicating that the therapeutic effect is likely achieved *via* upregulation of pro-angiogenic cytokines and downregulation of inflammation.

**Conclusion:**

This innovative cell therapy confers increased limb reperfusion, neovascularization, improved functional recovery, decreased muscle atrophy, and thus offers a potential therapeutic method for future clinical studies.

## Introduction

Critical limb ischemia (CLI), the most severe form of peripheral arterial disease (PAD), is the clinical syndrome that includes ischemic rest pain and tissue loss due to severely diminished perfusion to the affected limb. Of all patients with PAD, 11% will go on to develop CLI that confers a 50% 5-year mortality rate and a 70% 10-year mortality rate (1, 2). Risk factors such as smoking, obesity, and diabetes mellitus increase one's risk of developing PAD. Currently, the treatment modalities available for patients with CLI include medical risk reduction strategies as well as procedural revascularization such as endovascular angioplasty/stenting or bypass surgeries (3). Unfortunately, some patients are not surgical candidates or the revascularization may fail, which results in up to 30% of patients requiring limb amputation within 1 year of diagnosis, and is associated with a postamputation mortality rate up to 25% (4). Due to the reduced quality of life, significant financial costs, and poor survival outcomes among patients with CLI, vascular regenerative therapies that aim to increase limb salvage in these high-risk patients are a promising therapeutic option.

Therapeutic angiogenesis, the therapeutic development of new blood vessels, is an enticing regenerative concept to increase blood perfusion to the affected limb by which to promote tissue repair and regeneration. Gene therapy and cell therapy are two such strategies to employ therapeutic angiogenesis in localized tissues. Stem cell therapy, specifically the use of mesenchymal stem cells (MSCs), is advantageous due to its inherent ability to differentiate into multiple cell lineages required for tissue regeneration or secrete soluble factors or exosomes (paracrine actions) to promote neovascularization and tissue repair. Additionally, the immunomodulatory effects of MSCs may serve to dampen the negative effects of inflammation inherent in ischemic tissues (5, 6). Preclinical studies utilizing MSCs from the bone marrow, placenta, umbilical cord, and adipose tissue have demonstrated improved wound healing (7, 8) and ischemic hind limb reperfusion (9–12). However, treatments with unmodified MSCs in human clinical trials have shown modest translational effects on amputation-free survival (AFS), which has not always been shown to be superior to placebo (13–16). It is hypothesized that the limited therapeutic efficacy may be indicative of low potency in the selected cell populations administered (17–19). Thus, modifications of stem cells based on mechanistic understanding that enrolls regeneration pathways may boost their therapeutic activity resulting in more efficacious cell therapy for CLI.

One method of improving autologous MSC efficacy is by introducing genetic modifications that enhance the MSC's inherent ability to induce neovascularization and promote tissue repair. Our lab has demonstrated that E-selectin, an inducible cell-adhesion molecule that becomes upregulated in ischemic environments, is vital for the neovascularization and tissue repair processes (20, 21). We further demonstrated that coating MSCs, which normally do not express E-selectin, with nanocarrier complexed with soluble E-selectin, improved cell homing to the ischemic environment and improved repair and regeneration in a mouse model of hind limb ischemia (22). This study demonstrated that the MSCs overexpressing E-selectin are more biologically potent and can specifically interact with E-selectin ligands elevated on activated endothelial cells, thus actively contributing to the budding tip of vessel sprouts to promote angiogenesis. While this study was an important proof-of-concept, nanocarrier-coated MSC is not optimal for scale-up and human clinical trials. Moreover, there are potential applicability concerns related to the rapid internalization of nanocarrier bounded on the cell surface. Thus, we designed a viral vector utilizing the adeno-associated virus (AAV), which has been shown to be advantageous for genetic modifications and is a safe vector type already FDA-approved for gene therapy in humans (23, 24). This vector is utilized to transduce cells with resultant overexpression of E-selection on the cell's surface (herein referred to as supercharging), which we hypothesized would augment MSC's neovascularization and regeneration potential. In this work, we characterize biologic effects and explore the mechanisms of action.

## Materials and Methods

### Animals

The generation of the ROSA26-LacZ^+/−^ was performed by crossing C57BL/6 mice with B6; 129S-Gt(ROSA)26Sor/J mice (Jackson Laboratory, Bay Harbor, ME). Mice, hemizygous or homozygous for the ROSA26 retroviral insertion, display no distinguishing phenotype, lacZ is expressed in all tissues of the developing embryo and in most tissues of the adult mouse (25). Six to 8-week-old ROSA26-LacZ^+/−^ male/female mice were utilized for bone marrow extraction and 8–12 week-old male C57/BL6 mice were utilized for CLI-model creation and experimentation. The mice were maintained and bred under standard pathogen-free conditions, cared for and operated following the Policy on Use of Laboratory Animals, and all animal experiments were approved by the institutional animal care committee of the University of Miami (IACUC protocol 16-188). Euthanasia was performed *via* two methods as described by the IACUC protocol *via* CO^2^ gas inhalation and cervical dislocation.

### Murine Bone Marrow Extraction and Culture of MSCs

Bone marrow was harvested from the femurs of euthanized ROSA26-LacZ^+/−^ mice as previously described (26). Bone marrow cells were cultured in murine MSC MesenCult™ medium with supplement (STEMCELL Technologies, Vancouver, Canada). Non-adherent cells were removed every 96 h by changing the medium. MSCs were harvested at passage 1–2 for use in experiments. Cell growth was determined by different seeding densities and time points in triplicates.

### AAV Production

Full length murine genes of *E-selectin* and green-fluorescent protein (*GFP*) were inserted into multiple cloning sites in the pZac vector, respectively, and confirmed by gene sequencing. E-selectin-*ires*-eGFP/pZac and eGFP/pZac plasmids were then sent to University of North Carolina Gene Therapy Vector Core where AAV serotype 2/2 (AAV_2/2_) was packaged and preparations were performed per standard protocol using the 3-plasmid transfection into HEK293 cells (27). Quality assurance and control testing included qPCR quantification of AAV genomes, determination of infectivity titer, and tests for replication competent AAV (RCAAV) (27).

### Transduction of MSCs

Mesenchymal stem cells were transduced *ex vivo* with AAV_2/2_ (either E-selectin-*ires*-eGFP/AAV or eGFP/AAV, both under the control of the cytomegalovirus promoter) as previously described (28). Briefly, MSCs were trypsinized and plated in 100 mm cell culture plates at 5 × 10^5^ cell density with 10 ml Mesencult™ medium and were allowed to attach for 24 h before the virus was added directly to the medium at 5,000 viral genomes (VG)/cell. The media was changed after 12 h and every 96 h afterward for the remaining culture period. GFP transgene expression was assessed by immunohistology under fluorescence microscopy to determine transduction success and efficiency 3–4 days after transduction.

### Flow Cytometry

Mesenchymal stem cells were detached from culture dish using TrypLE™ express (Thermo Fisher Scientific, Waltham, MA) and collected in Eppendorf tubes. The cells were stained for MSC markers using anti-CD44, PE-Cy5 (BD Biosciences, Franklin Lakes, NJ, #553135), anti-CD73, BV605 (Biolegend, San Diego, CA, #127215), anti-CD105, AF647 (Biolegend, San Diego, CA, #120405), and anti-E-selectin/CD62E, PE (BD Biosciences, Franklin Lakes, NJ, #553751) to assess E-selectin levels. The cells were analyzed on a on a FACSAria II cell sorter using FACSDiva Version 6.1.1 (BD Biosciences) or FlowJo Version 7.6.4 (TreeStar) software.

### Creation of a Mouse CLI Model

Creation of hind limb ischemia mouse model was performed as previously described (29). Briefly, 8–10-week-old male mice underwent femoral artery ligation (FAL). To do this, the mice were anesthetized with ketamine 100 mg/kg and xylazine 10 mg/kg Intraperitoneal (IP); then, hair was sheared from the hind limbs and an incision was made to expose the femoral sheath. The femoral artery and vein were dissected and separated from the femoral nerve distally at the sapheno-popliteal bifurcation and proximally below the inguinal ligament. Both artery and vein were ligated at these two locations with 7-0 silk sutures and the intervening segment between sutures severed with a single incision (Supplementary Figure 1A). The mice were injected with 100 μl of 5 × 10^5^ MSC^GFP^, MSC^E−selectin−GFP^, or PBS (vehicle) at 4 points (25 μl/point) in the thigh muscle of the surgical limb after ligation, postoperative day (POD) 1 and POD 2. Finally, the skin incision was closed using 7-0 silk suture in a continuous fashion. A single dose of sustained-release buprenorphine (ZooPharm, 1.0 mg/kg) was subcutaneously administered for postoperative analgesia.

### Laser Doppler Imaging

Laser Doppler imaging (LDI) was performed using the LDI2-HR System from Moor Instruments (Wilmington, DE). LDI was performed on the ligated and non-ligated leg preoperatively and immediately after FAL, and subsequently on POD 7, 14, and 21. As previously detailed, the mice were lightly anesthetized (30, 31) and placed on a heating pad to maintain their core body temperature at 37°C. Image of the plantar foot was of particular interest since this area most effectively demonstrates reperfusion on LDI. Hind limb reperfusion index was defined as a ratio of ligated to non-ligated leg for each individual mouse.

### Live Animal DiI Perfusion and Confocal Laser Scanning Microscopy

The lipophilic carbocyanine dye DiI (1,1′-dioctadecyl-3,3,3′,3′-tetramethylindocarbocyanine perchlorate (Invitrogen, Carlsbad, CA, #D282) was used for vascular perfusion and staining as previously described (29, 32, 33). Briefly, intramyocardial perfusion of filtered PBS followed by 10 mL of DiI and lastly 10% neutral formalin for tissue fixation was performed on POD 21. The area of interest was the footpad, given our interest in distal hind limb reperfusion. The skinned murine footpad was compressed between two glass micro slides using binder clips for adequate imaging with the Zeiss LSM 510 confocal laser scanning microscope. Z-series obtained from confocal imaging were reconstructed into 3-dimensional images and analyzed using Image J. The intricate network of vessels regenerated in the foot was captured, quantified, and represented by mean capillary density (percentage of volume of total DiI-stained vessels per area within foot) for each mouse.

### Histology

Immunofluorescent staining and H&E were performed as per a previously published protocol (34, 35). Slides of tissue sections were deparaffinized, hydrated, washed, and blocked with protein blocking serum (Dako, Carpinteria, CA) for an hour at 25°C. To determine the MSC location within thigh tissue sections, the slides were incubated with anti-GFP (Invitrogen, Carlsbad, CA, #A21312) 1.5 μg/mL in protein blocking serum for 12 h at 4°C. The slides were then washed with 0.1% Tris-buffered saline and polysorbate-20 (TBST) prior to 4′,6-diamidino-2-phenylindole (DAPI) staining (Vector Laboratories, Inc., Burlingame, CA) for nuclei visualization. Confocal laser scanning microscope using 20–40 × magnification was used to image immunofluorescence. For quantification of presence of GFP^+^ MSCs, at least 10 random ×40 magnification fields per section were blindly scored and/or measured with Image J or Adobe Photoshop CC. To determine muscular atrophy in the ligated limbs of mice after FAL, H&E staining was performed on paraffin-embedded transverse muscle tissue sections of the treatment groups as previously described (36). Images were then acquired *via* 20X magnification and 10 random myocytes in each field were measured circumferentially based on actual measurement reflected by size bar and total surface area was calculated (μm^2^).

### Treadmill Exhaustion Test

Exercise treadmill testing was performed on a rodent treadmill as previously described (37, 38) (Columbus Instruments, Columbus, OH, model: Exer3/6) 21 days after FAL to determine the degree of hind limb functionality and recovery after treatment. In this study, our protocol started with a 10° slope at a speed of 10 meters/minute for 5 min. The mice were placed on a horizontal treadmill, and the speed was increased by 5 meters/minute every 5 min until a maximum speed of 30 meters/min was reached. The mice were allowed to run until exhaustion occurred, which was defined as spending 5 consecutive seconds on the stimulus pad or visiting the stimulus pad a total of 40 times. The total distance (m) traversed prior to exhaustion was then recorded for each mouse.

### Gene Expression Analysis by PCR Array

The Murine Angiogenesis RT^2^ Profiler™ PCR array quantitatively profiles the expression of 84 genes involved in angiogenesis (Qiagen, Hilden, Germany, #PAMM-024Z). Total RNA was extracted from the cells and tissues using Trizol^®^ (Invitrogen, Carlsbad, CA, # 15596026) and cDNA was synthesized using RT^2^ First Strand Kits (Qiagen, Hilden, Germany, #330401). PCR array was carried out according to the manufacturer's protocol. The threshold cycle (Ct) values were used to plot a standard curve. All samples were normalized to the relative levels of GAPDH, and results are expressed as fluorescence intensity in relative levels (cultured MSC^E−selectin−GFP^ vs. MSC^GFP^ and ischemic tissue treated with MSC^E−selectin−GFP^ vs. MSC^GFP^).

### Biodistribution of Engrafted MSCs

To detect the biodistribution of locally engrafted MSCs, total RNA was extracted from various tissues, including treated ischemic limbs, lungs, and livers, using Trizol^®^ (Invitrogen, Carlsbad, CA, # 15596026) and cDNA was synthesized using RT^2^ First Strand Kits (Qiagen, Hilden, Germany, #330401). PCR was then performed to detect the presence of GFP (primer: 5′-AAGCTGACCCTGAAGTTCATCTGC-3′, 5′-CTTGTAGTTGCCGTCGTCCTTGAA-3′), GFP/AAV viral DNA was utilized for a positive control and vehicle muscle tissue was negative control. After PCR, gel electrophoresis in 2% agarose gel with ethidium bromide was performed at 90 mV for 40 min. Images of gel were acquired using the Gel Doc XR™ (Bio Rad, Hercules, CA).

### Statistical Analysis

Data are presented as mean ± SD. Two-tailed Student's *T*-tests were performed to compare the means of two groups and one-way ANOVA was performed to compare the means of three or more groups, with *post-hoc* Tukey Simultaneous Tests for Differences of Means. An alpha value of *P* < 0.05 was interpreted to denote statistical significance.

## Results

### Characterization of MSCs With Effective Viral Transduction Utilizing AAV

**Figure 1:**
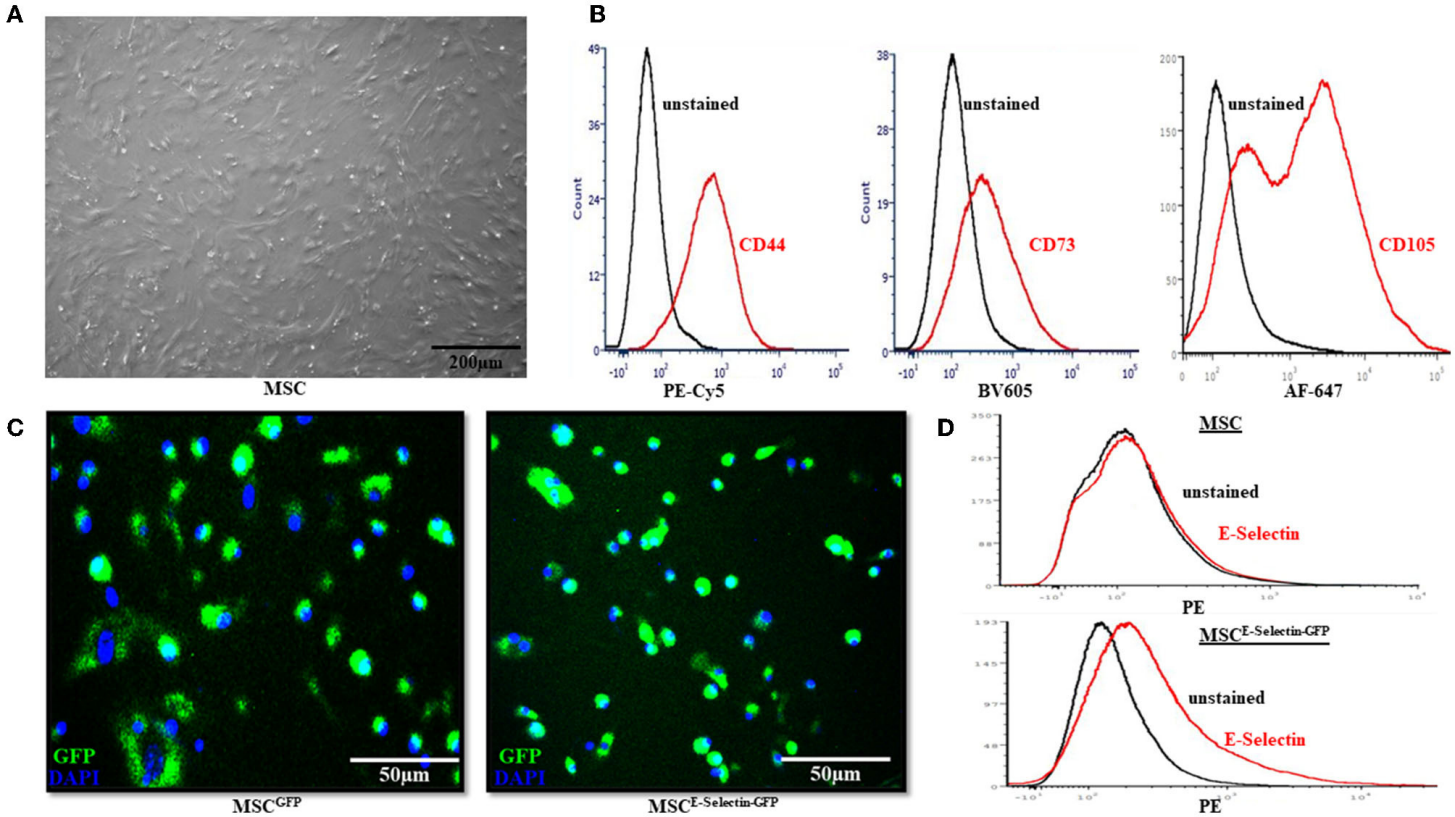
Characterization of Mesenchymal Stem Cells (MSC). **(A)** MSC demonstrated fibroblast-like morphology in the growth medium and were adherent to the cell culture dish. **(B)** Flow cytometry revealed the native MSC had expression of cell-surface molecules CD44, CD73, and CD105. **(C)** After cells were transduced with either GFP/AAV or E-selectin-*ires*-eGFP/AAV, successful cell transduction is demonstrated by expression of GFP under confocal microscopy. **(D)** Flow-cytometry demonstrates a lack of E-selectin expression in untransduced MSC (top) and a gain of E-selectin expression after transduction has been performed (bottom).

To ensure that the MSCs were supercharged as intended, several *in vitro* characteristics were assessed before *in vivo* viral transduction took place. The MSCs demonstrated fibroblast-like morphology and were adherent to the cell-culture plate (Figure 1A). Flow cytometry demonstrated positive expression of MSC markers CD44, CD73, and CD105 (Figure 1B). After transduction with either E-selectin-*ires*-eGFP/AAV or eGFP/AAV, fluorescence microscopy was utilized to ensure transgene expression of GFP. Nuclei were stained with DAPI (Figure 1C). Almost all cells were successfully transduced by AAV (overlapping of GFP and DAPI), indicating a near 100% transduction efficiency under the utilized transduction conditions. Flow cytometry demonstrated that native MSCs do not express E-selectin (Figure 1D, top), while the MSCs transduced with E-selectin-*ires*-eGFP/AAV express elevated levels of E-selectin (Figure 1D, bottom).

### Supercharging MSCs With AAV-Mediated E-Selectin Expression Enhances the Therapeutic Phenotype

**Figure 2:**
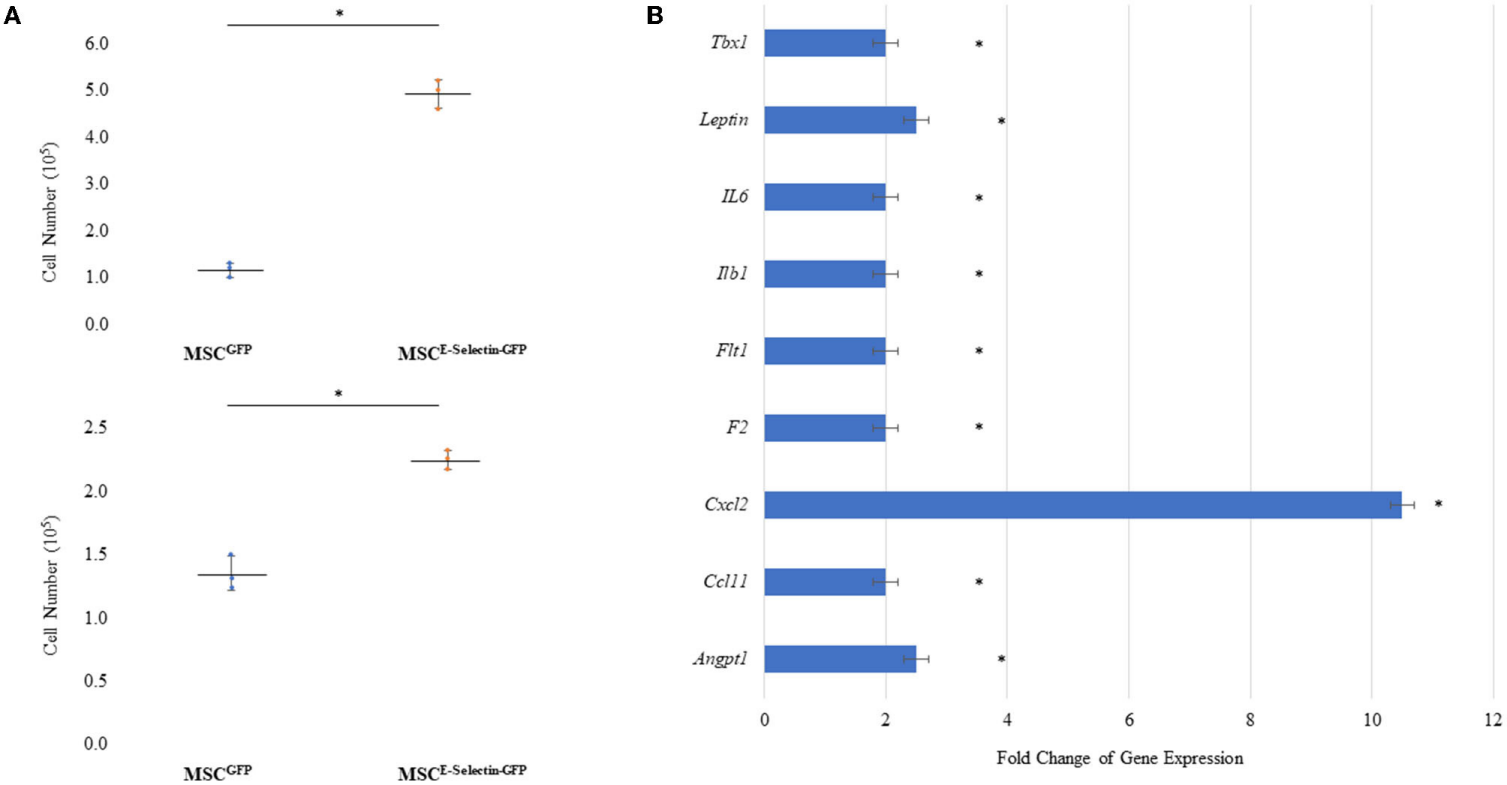
Characteristics of MSC Supercharged with E-selectin. **(A)** Cell growth was enhanced post-transduction with E-selectin in either (top) plating density of 0.5 × 10^5^ or (bottom) 0.1 × 10^5^ when compared to control (mean ± SD of triplicates for each seeding density, *P < 0.05 by Student's *T*-test). **(B)** Expression of several chemokine/cytokine mRNA was significantly higher in the MSC^Eselectin−GFP^ vs. MSC^GFP^ measured by quantitative PCRArray. Equal portions of RNA from three plates in each group were utilized to perform reverse transcription reaction and each experiments was repeated for three times (mean ± SD of fold change MSC^E−selectin−GFP^ vs. MSC^GFP^, *P < 0.05 by Student's *T*-test).

To determine whether supercharging MSCs with E-selectin resulted in a superior therapeutic phenotype, cell proliferation and gene expression profiling were performed. MSC^E−selectin−GFP^ was observed to have increased cell-culture numbers upon harvest from culture plates. We utilized MSC^E−selectin−GFP^ and MSC^GFP^ (control) to compare their growth potentials at two different seeding densities (0.1 × 10^5^ and 0.5 X 10^5^ cells/well). We demonstrate herein that MSC^E−selectin−GFP^ has increased cell proliferation within cell culture compared with MSC^GFP^ at both seeding densities (Figure 2A, *P* < 0.05). We then sought to determine genetic expression changes *via* PCR array profiling in MSC^E−selectin−GFP^ vs. MSC^GFP^. To do this, we extracted RNA from cultured MSC^E−selectin−GFP^ and MSC^GFP^, and performed RT^2−^Profiler PCR array of 84 genes involved in angiogenesis. This analysis demonstrated that compared with MSC^GFP^, MSC^E−selectin−GFP^ had significantly upregulated genetic expression of 9 genes (*Tbx1, Leptin, IL6, Ilb1, Flt1, F2, Cxcl2, Ccl11, Angpt1*, Figure 2B, *P* < 0.05), Table 1.

**Table 1:** Genes upregulated in E-selectin supercharged mesenchymal stem cells (MSCs) (compared to MSC^GFP^) and their previously described functions.

**Symbol**	**Gene name**	**GenBank**	**Function**
Tbx1†	T-box 1	NM_011532	Controls vascular smooth muscle and extracellular matrix investment in great vessel remodeling (39)
*Lep*†	Leptin	NM_008493	Regulates energy homeostasis (40)
*IL6*	Interleukin-6	NM_031168	Mediates the immune system and regenerative processes (41)
*Ilb1*	Interleukin-1β	NM_008361	Mediates inflammation and lymphocyte activation (42)
*Flt1*	Fms Related Receptor Tyrosine Kinase 1	NM_010228	Acts as a cell-surface receptor for VEGFA, VEGFB, and PGF and regulates cell migration along with post-natal angiogenesis (43–45)
*F2*†	Coagulation Factor II	NM_010168	Involved in blood homeostasis and wound healing (46)
*Cxlc2*†	Chemokine (C-X-C motif) ligand 2	NM_009140	Plays a role in improved cellular trafficking and engraftment (47)
*Ccl11*	C-C Motif Chemokine Ligand 11	NM_011330	Promotes accumulation of eosinophils (48)
*Angpt1*†	Angiopoietin 1	NM_009640	Promotes endothelial cell survival for angiogenesis (49)

*Table listing full name of upregulated genes with the **†** mark indicating the genes also upregulated in vivo within the ischemic tissue injected with supercharged MSC^E−selectin−GFP^ vs. the ischemic tissue injected with control MSC^GFP^*.

*VEGFA, vascular endothelial growth factor a; VEGFB, vascular endothelial growth factor B; PGF, placental growth factor*.

### Treatment With MSC^E-Selectin-GFP^ Improves Reperfusion Index in Murine Model of Hind Limb Ischemia

**Figure 3:**
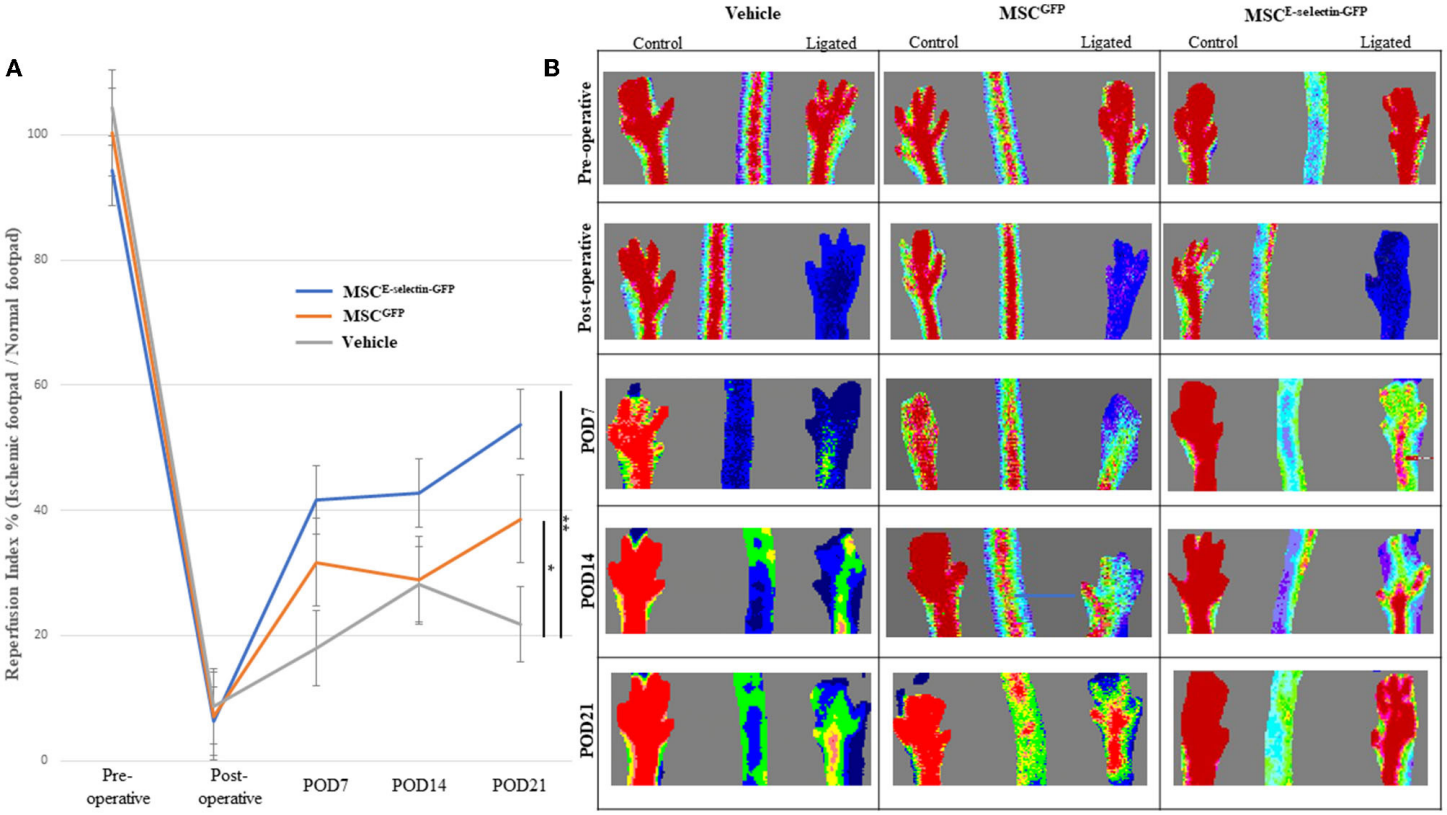
MSC^E−selectin−GFP^ treatment increases ischemic limb reperfusion. **(A)** The quantitative analysis of limb reperfusion measured by LDI (reperfusion index % = ischemic ligated footpad/normal control footpad, mean ± SD; **P < 0.01 by ANOVA and *P < 0.05 by *post-hoc* analysis, Vehicle *n* = 6, MSC^GFP^ *n* = 20, MSC^E−selectin−GFP^ *n* = 18). **(B)** Representative images of hindlimb reperfusion by LDI obtained preoperatively, immediately after FAL (postoperative), and on postoperative days (POD) 7, 14, and 21.

We further evaluated the overall effect of MSC^E−selectin−GFP^ therapy on the extent of hind limb ischemia, *in vivo*. Distal hind limb ischemia was surgically induced in mice that were then randomly separated into three groups, each treated with either MSC^E−selectin−GFP^ (*n* = 18), MSC^GFP^ (*n* = 20), or PBS (vehicle, *n* = 6). LDI measurement reflects the vascular perfusion at the skin and subcutaneous tissues of the mice hind limbs, which serves as an overall reflection of the hind limb revascularization process. LDI measurements performed preoperatively and immediately postoperative were comparable between all groups (Figure 3). By POD 21, the mice treated with MSC^E−selectin−GFP^ demonstrated statistically significantly increased distal hind limb blood flow, as evidenced by increased reperfusion index (ligated limb / non-ligated limb) on Doppler imaging when compared with mice treated with either MSC^GFP^ or vehicle (Figure 3A, *P* < 0.01). *Post-hoc* analysis demonstrated that treatment with MSC^GFP^ resulted in superior reperfusion index when compared with vehicle (*P* < 0.05, Figure 3A). Overall, these results reveal that there is an increased level of reperfusion in the ischemic hind limb treated with both types of MSCs but the effect is significantly more potent with the E-selectin supercharged MSCs.

### MSC^E-Selectin-GFP^ Therapy Increases Vessel Density in the Murine Ischemic Footpad

**Figure 4:**
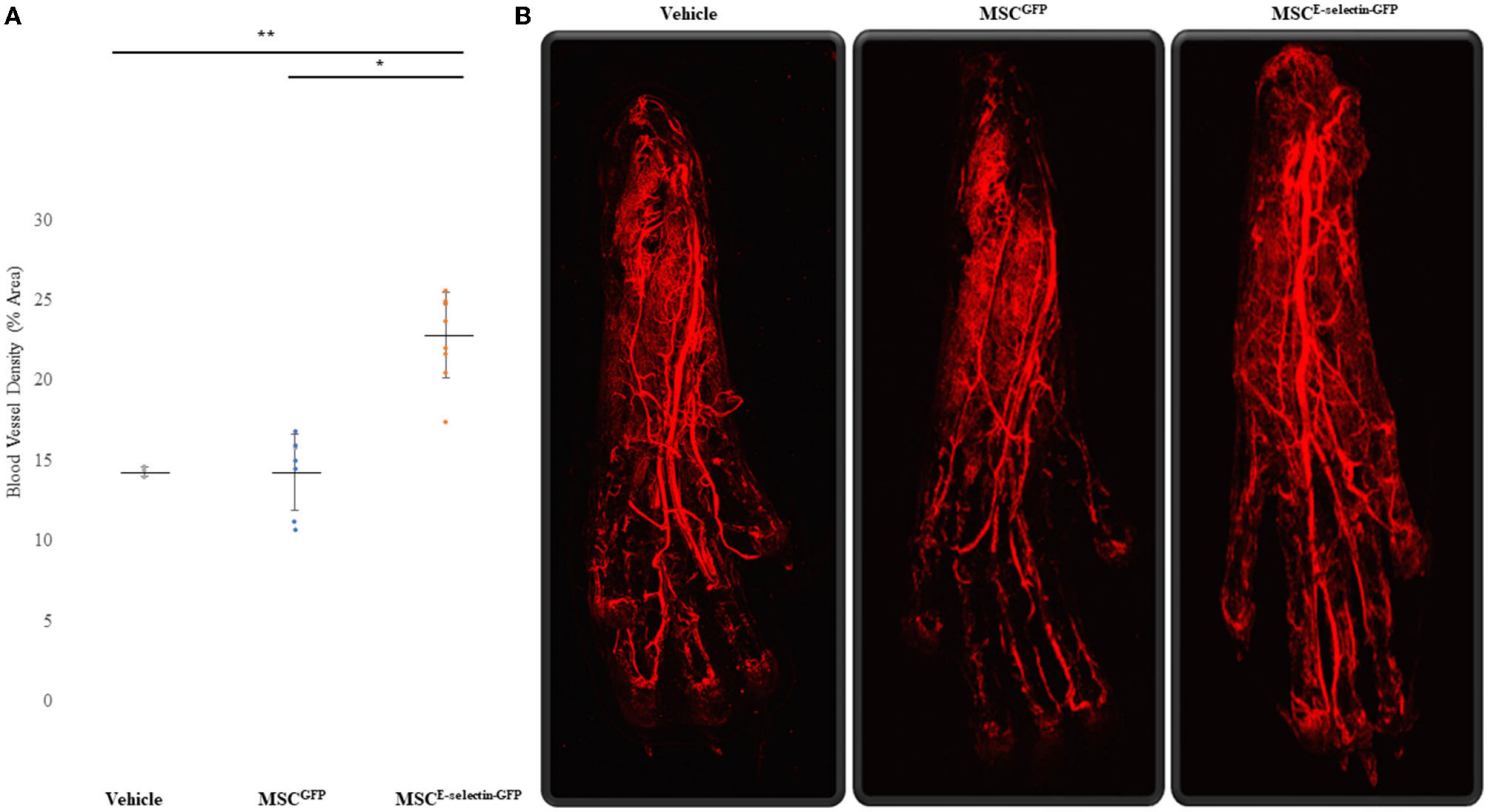
Improved Blood Vessel Density of the Ischemic Distal Footpad after treatment with MSC^E−selectin−GFP^. **(A)** Qualitative analysis of the distal toe vessel density (% area, mean ± SD, **P < 0.01 *via* ANOVA, *P < 0.05 *post-hoc* Tukey Simultaneous Tests for Differences of Means, Vehicle treatment *n* = 3, MSC^GFP^ *n* = 8, MSC^E−selectin−GFP^ *n* = 9). **(B)** Representative images of Z-stacked images of the ischemic footpads after DiI perfusion *via* Confocal laser microscopy.

To determine whether treatment with MSC^E−selectin−GFP^ can increase postnatal neovascularization, 21 days after FAL live animal whole-body DiI perfusion followed by confocal laser scanning photography of the ischemic footpad was performed to evaluate blood vessel density within the murine footpad in mice treated with MSC^E−selectin−GFP^ (*n* = 9), MSC^GFP^ (*n* = 8), or PBS (vehicle, *n* = 3). There was a statistically significant difference in the number of vasculature, including capillaries, in the ischemic footpads of the mice treated by MSC^E−selectin−GFP^ as opposed to treatment with MSC^GFP^ or vehicle (Figure 4, *P* < 0.01). Quantitatively, the mean capillary density within the ischemic footpad of mice treated with MSC^E−selectin−GFP^ was 23%, while MSC^GFP^ and vehicle were 14 and 14%, respectively (Figure 4A). Taken together, these data demonstrate that treatment with MSC^E−selectin−GFP^ induces increased vessel density in the footpad in mice with surgically induced hind limb ischemia.

### MSC^E-Selectin-GFP^ Therapy Improves Limb Functional Performance With Improved Exercise Tolerance and Decreased Hind Limb Skeletal Muscle Atrophy

**Figure 5:**
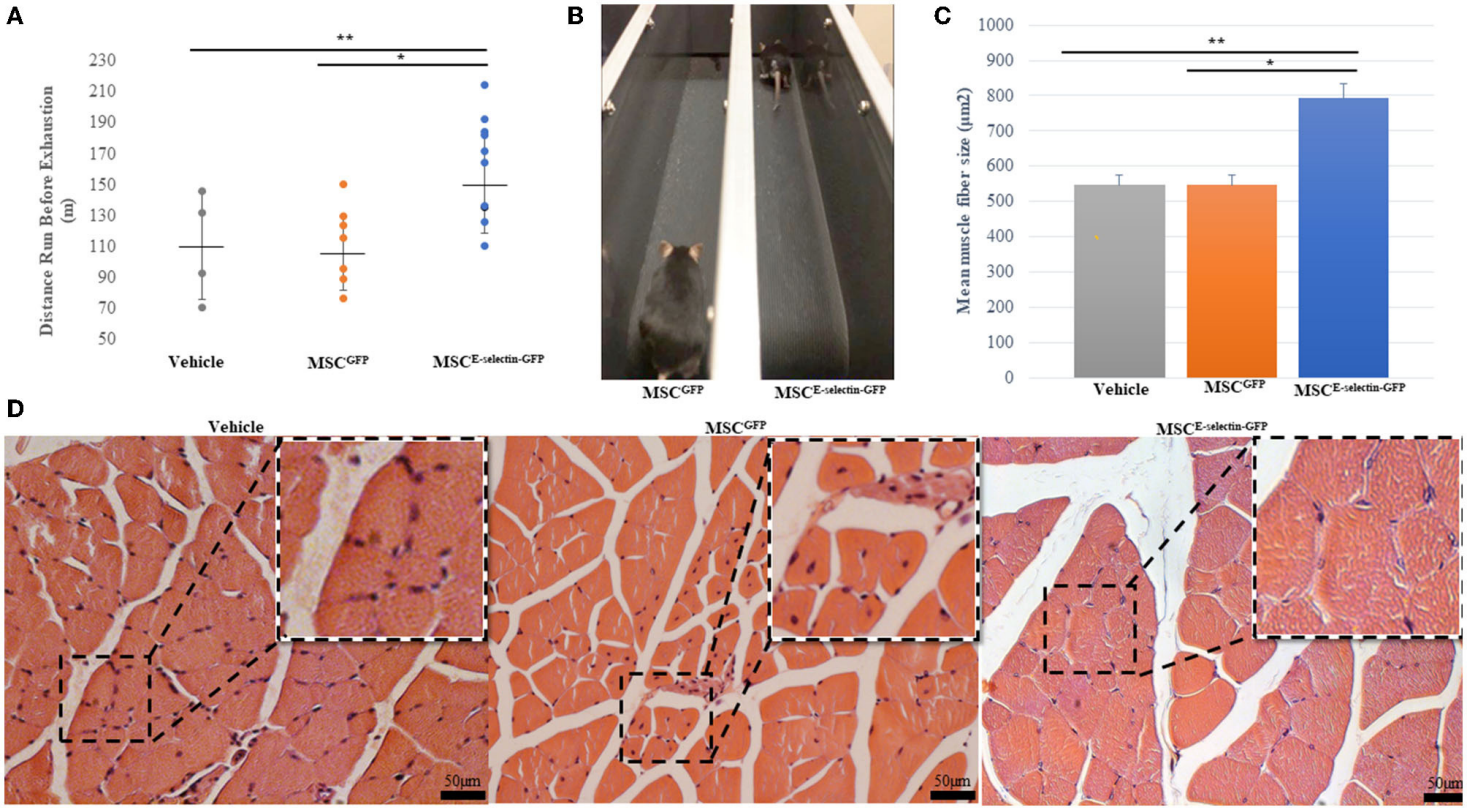
Improved functional and pathologic recovery in ischemic muscles after treatment with MSC^E−selectin−GFP^. **(A)** Quantitative analysis of distance ran (m) prior to exhaustion in treatment groups (Vehicle = 4, MSC^GFP^ = 7, MSC^E−selectin−GFP^ = 10), **P < 0.01 *via* ANOVA). **(B)** Representative image of MSC^GFP^ vs. MSC^E−selectin−GFP^ on treadmill. **(C)** Qualitative analysis of the mean myofiber size of the ischemic calf muscle, data are based on 5 section/sample/mouse (μm^2^, mean ± SD, **P < 0.01 *via* ANOVA, *P < 0.05 *post-hoc* Tukey Simultaneous Tests for Differences of Means, Vehicle treatment *n* = 3, MSC^GFP^ treatment *n* = 3, MSC^E−selectin−GFP^ treatment *n* = 3). **(D)** Representative images of transverse H&E sections of ischemic calf muscle in Vehicle, MSC^GFP^, and MSC^E−selectin−GFP^.

To assess whether cell therapy treatment with MSC^E−selectin−GFP^ would confer increased limb functional performance and decreased muscular atrophy, the treadmill exhaustion test was performed 21 days after induced ischemia in all treatment groups. The treadmill exhaustion test allowed mice in all treatment groups to run to exhaustion. The mice treated with MSC^E−selectin−GFP^ had significantly increased exercise tolerance as evidenced by longer mean distance ran before exhaustion when compared with either MSC^GFP^ or vehicle (Figure 5A, *P* < 0.01). The mice treated with MSC^E−selectin−GFP^ ran a mean distance of 162 meters before exhaustion while the mice treated with MSC^GFP^ and vehicle ran mean distances of 111 and 110 m, respectively. The mice treated with MSC^E−selectin−GFP^ consistently stayed on the treadmill and furthest away from the adverse stimuli grid (Figure 5B). H&E sections of ischemic calf muscles harvested at POD 21 were examined and less muscle atrophy was noted in the mice treated with MSC^E−selectin−GFP^ compared with those treated by MSC^GFP^ or vehicle. The muscle fibers of mice treated with MSC^E−selectin−GFP^ had a significantly larger cross-sectional surface area when compared with mice treated with MSC^GFP^ or vehicle (Figures 5C,D). The muscle fibers in mice treated with MSC^E−selectin−GFP^ had a mean muscle fiber size of 793 μm^2^, while mice treated with MSC^GFP^ and vehicle had mean muscle fiber sizes of 526 and 546 μm^2^, respectively (Figure 5C, *P* < 0.01). Overall, our data demonstrate that cell therapy treatment with MSC^E−selectin−GFP^ improves limb functional recovery while mitigating skeletal muscle atrophy in this murine model of induced CLI.

### *In vivo* Tissue Gene-Expression Profile Changes After Treatment With MSC^E-Selectin-GFP^

**Figure 6:**
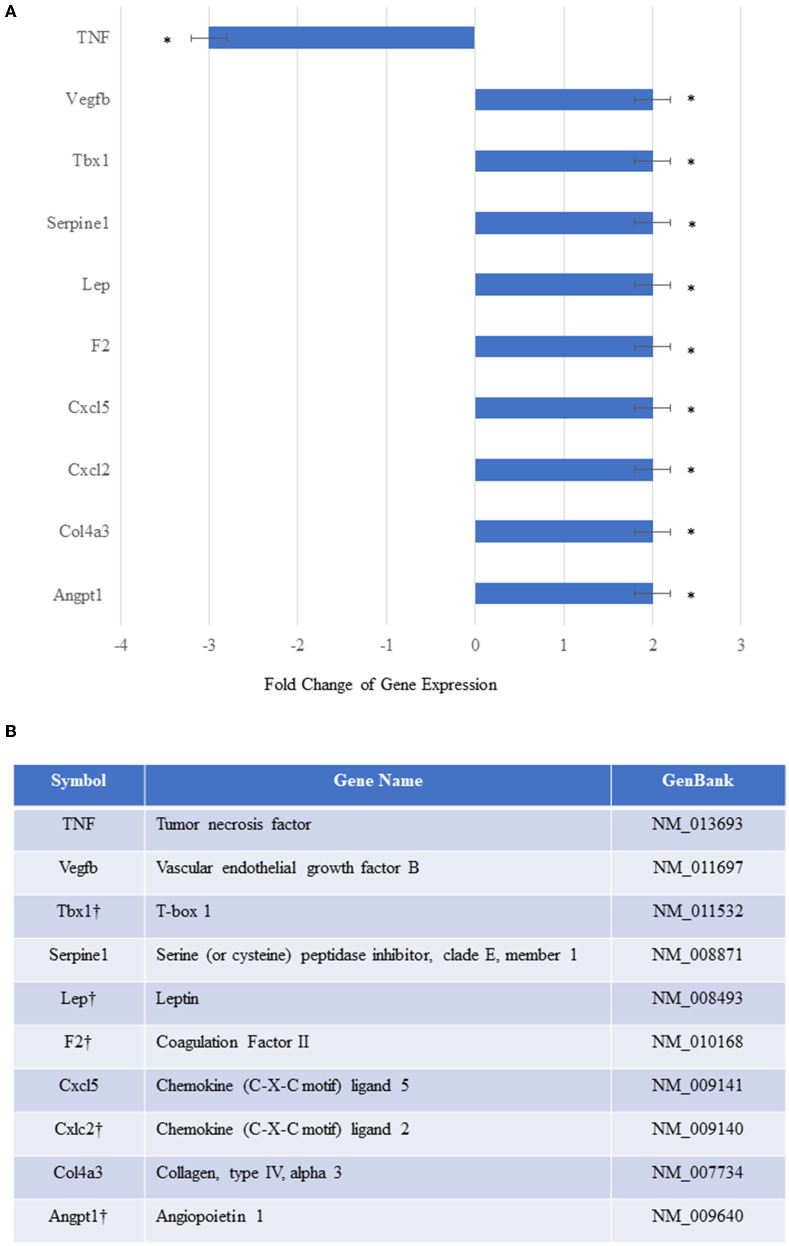
*In vivo* relative gene expression changes in the ischemic thigh tissue of mice treated with MSC^Eselectin−GFP^ vs. MSC^GFP^. **(A)** Expression of several chemokine/cytokine mRNA was significantly higher in the ischemic thigh muscle treated with MSC^Eselectin−GFP^ vs. MSC^GFP^ measured by quantitative PCRArray by post-operative day 21. However, tumor necrosis factor (TNF) was significantly downregulated. Equal portions of RNA from three mice in each treatment group were utilized to perform reverse transcription reaction. Each experiment was repeated three times and data are presented as fold-change of treatment/control (*P < 0.05 *via* Student's *T*-test). **(B)** Table listing full name of upregulated genes with the † mark indicating the genes also upregulated *in-vitro* within the injected supercharged MSC^E−selectin−GFP^ vs. the control MSC^GFP^.

To elucidate the mechanisms underlying MSC^E−selectin−GFP^ therapy, we examined with an array of genes involved in angiogenesis and other functions. We utilized an *RT*^2^*-PCR-Array* analysis of 84 angiogenic and inflammation signaling genes to investigate the genetic expression changes seen in the ischemic limb tissues treated with MSC^E−selectin−GFP^ vs. MSC^GFP^ 21 days after treatment. We demonstrated that the ischemic muscle tissues treated with MSC^E−selectin−GFP^ had significantly upregulated genetic expression of 9 genes (*Vegfb, Tbx1, Serpine1, Leptin, F2, Cxcl5, Cxcl2, Col4a3, and Angpt1*), and significantly downregulated expression of *TNF* gene (Figure 6A, *P* < 0.05). Four of those tissue-level genes were also upregulated *in vitro* by the MSC^E−selectin−GFP^ utilized to treat the ischemic limbs: *Tbx1, Leptin, F2*, and *Cxcl2* (Figure 6B). Levels of several angiogenic genes, including VEGF, HGF, and PDGF, were unchanged in the culture MSC^E−selectin−GFP^ compared with MSC^GFP^ *in vitro* according to *RT*^2^*-PCR-Array* (Supplementary Table 2). Thus, *Tbx1, Leptin, F2*, and *Cxcl2* upregulated in treated ischemic limb tissues may be derived directly from the engrafted MSC^E−selectin−GFP^, while likely the upregulation of the other 5 genes (*Vegfb, Serpine1, Cxcl5, Col4a3, and Angpt1*) and downregulation of *TNF* gene in ischemic limb tissues are indirect effects of the supercharged MSC cell therapy, from activation of secondary cell-cell and paracrine signaling cascades. These data suggest that the therapeutic effect of MSC^E−selectin−GFP^ may be mediated through both direct and indirect mechanisms from primary and secondary signaling cascades.

### Determining the Persistence, Biodistribution, and Potential Toxicity of MSCs Engrafted in the Ischemic Hind Limb

**Figure 7:**
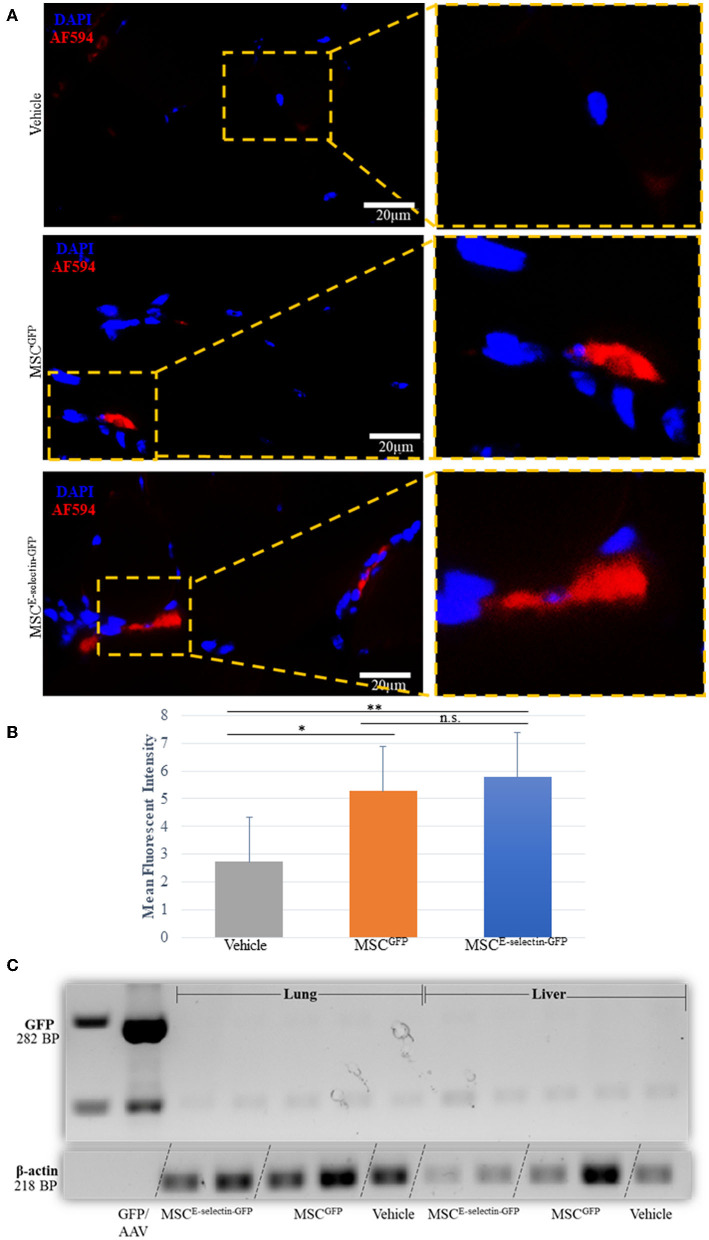
Determining the fate of MSC injected as treatment for hindlimb ischemia 3-weeks after injection into thigh muscle. **(A)**. Representative images of immunohistochemistry co-staining with DAPI and GFP (AF-594), demonstrating that a few of the injected MSC survive at 3-weeks (POD 21). **(B)** Quantitative analysis of mean fluorescent intensity (MFI) in ischemic thigh muscle tissue sections stained for GFP demonstrates significant increased MFI between MSC^E−selectin−GFP^, MSC^GFP^, and Vehicle (*n* = 30 sections/group, **P < 0.001, *P < 0.01, n.s. = not significant *via* ANOVA with *post-hoc* Tukey Simultaneous Tests for Differences of Means) **(C)**. Representative PCR gel electrophoresis of mice lung and liver tissue demonstrating lack of GFP expression (positive control located at 282BP) and positive β-actin for MSC^E−selectin−GFP^, MSC^GFP^, and Vehicle (at 218BP).

We performed immunofluorescent staining of the tissue sections treated with either MSC^E−selectin−GFP^, MSC^GFP^, or vehicle to assess the persistence of MSCs within the ischemic limb tissues harvested at POD 21. Very rare double positive (DAPI^+^/GFP^+^) cells were found within the treated ischemic limb tissues as evidenced by the representative images (Figure 7A) and quantification (Figure 7B), indicating that a few cells survive at POD 21. To determine the systemic biodistribution of MSCs engrafted in the ischemic hind limb of mice, we employed PCR analysis of remote organs. Lungs and liver represent two organs for engrafted MSCs to spread easiest *via* veins and arteries. These were harvested at POD 21 to detect the expression of *GFP* gene as both MSC^E−selectin−GFP^ and MSC^GFP^ express GFP. First, we extracted total RNA from treated ischemic limb tissues, lungs, and livers, and utilized the cDNA generated by reverse transcription of mRNA for PCR analysis. We demonstrate a positive band (282 bp) within the viral DNA (GFP/AAV, positive control), and an absence of bands for distant organs (liver and lungs) in the treated mice (Figure 7C). Additionally, blood tests were performed to examine for potential systemic toxicity of MSC^E−selectin−GFP^ therapy. Among all three treatment groups, there was no leukocytosis, anemia, or alterations in hepatic or renal metabolism (Supplementary Table 1), while there was a trend toward higher AST levels in the vehicle group. Overall, these data demonstrate that the described cell-based therapy is generally not toxic. Most engrafted MSCs disappear by 21 days, which suggests that the therapeutic effect is achieved largely by early direct and indirect paracrine mechanisms. The fact that only a few cells survive in treated limb tissues may also implies that this MSC therapy has a lower theoretical long-term risk for tumorigenesis. Moreover, locally engrafted MSCs do not spread to distant organs (lungs and liver) and do not appear to have systemic toxic effects, indicating early preclinical evidence of biosafety with the MSC^E−selectin−GFP^ therapy.

## Discussion

Mesenchymal stem cells throughout the last few decades have demonstrated strong regenerative and therapeutic potentials and have raised a great deal of hope for patients suffering from occlusive PAD. Their utilization as a treatment for CLI has demonstrated feasibility and efficacy in preventing disease progression, while very modest efficacy has been observed for all-cause mortality and amputation rates. However, clinical trials to date have employed unmodified MSCs, which may suggest that autologous MSCs, especially those from older, diseased patients, require additional modification to increase therapeutic efficacy (17, 50). Herein, we utilized a novel strategy of incorporating the pro-angiogenic (34, 51), and pro-healing (29) effects of E-selectin with the immunomodulatory and pro-regenerative properties of MSCs (8, 52) to improve the potential therapeutic efficacy of a stem-cell-based approach to CLI. In this study, we utilized the AAV to induce overexpression of E-selectin on the surface of MSCs. This creates a cell-based product that does not occur in nature, as indicated by the absence of E-selectin on the surface of control MSCs. Genetically modified MSCs have been employed in regenerative medicine, particularly in vascular regenerative preclinical studies to enhance the pro-angiogenic properties inherent in MSCs. Most investigations employ genetic modifications to increase angiogenic growth factors such as stromal cell-derived factor 1(SDF-1α) (53), platelet-derived growth factor (P-DGF) (47), vascular endothelial growth factor (VEGF) (40), and hepatocyte growth factor (HGF) (41), among others (39). These studies have demonstrated improved efficacy when compared with control MSCs, thus indicating MSC efficacy may be enhanced *via* genetic modifications. To our knowledge, this is the first attempt to use an adhesion molecule as the payload for a gene-modified cell-based approach in CLI.

We demonstrated improved therapeutic angiogenesis in hind limb ischemia *via* intramuscular injections of MSC^E−selectin−GFP^ in comparison to MSC^GFP^. We then further investigated the genetic expression resulting *in vitro* and *in vivo*. Our data demonstrate that treatment with MSC^E−selectin−GFP^ not only improved tissue angiogenesis as evidenced by improved neovascularization on LDI and increased distal blood vessel density on DiI perfusion of murine footpads, but also improved muscular functional recovery and mitigated skeletal muscular atrophy in the ischemic murine hind limb. PCR array gene profiling suggests that treatment with MSC^E−selectin−GFP^ had increased direct and downstream angiogenic chemokines/cytokines in addition to tissue downregulation of TNF, which likely assists in the modulation of the angiogenic response, dampens inflammation, and reduces the ischemia-induced tissue damage.

We utilized a AAV vector to transfer the E-selectin gene into MSCs, which do not normally overexpress E-selectin, to create a gene-modified cell-based therapy that could be applied to CLI. Past attempts at therapeutic angiogenesis utilizing viral vectors have been through gene therapy approaches with recombinant adenovirus (γAd) (42–46). Herein, we utilize the recombinant AAV (γAAV) due to its safety profile (48), high efficiency transduction in MSCs (28), and AAV transduction, which has gained approval for gene therapy from the Federal Drug Administration (FDA) in other disease processes (23, 24). The AAV vector used in this work is a non-pathogenic parvovirus that has the ability to transduce non-dividing and dividing cells with efficient gene expression and with minimal immunologic reaction (49). We demonstrated high-efficiency viral transduction and effective expression of the E-selectin transgene. Under the condition of 5 × 10^3^ viral genome (VG)/cell, we were able to achieve almost 100% transduction efficiency without cellular toxicity. Achieving high transduction efficiency for MSCs *in vitro* with relatively low multiplicity of infection (MOI) of AAV marks this viral vector as a useful tool for gene-modified cell-based stem cell therapy. Supercharging the MSCs with E-selectin resulted in a phenotypic characteristic that increased their therapeutic potential. Specifically, MSC^E−selectin−GFP^ demonstrated increased growth within the cell-culture plates and upregulation of pro-angiogenic genes involved in the production and secretion of chemokines and cytokines necessary for blood vessel development and tissue repair.

Supercharged MSC^E−selectin−GFP^ overexpressed 9 angiogenic genes *in vitro* when compared to MSC^GFP^. These 9 genes involved in angiogenesis also have multiple additional functions. Chemokine (C-X-C) motif, Ligand 2 (*Cxcl-2*), is known to play a role in improved cellular trafficking and engraftment (54), Leptin regulates energy homeostasis (55), interleukin-6 (*IL-6*) mediates the immune system and regenerative processes (56), while T-box transcription factor-1 (*Tbx1*) regulates arterial development (57). Interleukin-1β (*Il1b*) mediates inflammation and lymphocyte activation (58), Fms-related Receptor Tyrosine Kinase 1 (*Flt1*) acts as a cell-surface receptor for vascular endothelial growth factor A (VEGFA), vascular endothelial growth factor B (VEGFB), placental growth factor (PGF), and regulates cell migration along with postnatal angiogenesis (59–61), and coagulation factor II (*F2*) is involved in blood homeostasis and wound healing (62). C-C motif Chemokine Ligand 11 (*Ccl11*) promotes accumulation of eosinophils (63), and Angiopoietin-1 (*Angpt1*) promotes endothelial cell survival for angiogenesis (64). MSCs have known immunomodulatory effects (65). Pro-inflammatory MSCs (MSC1) produce cytokines such as IL-1β, IL-6, IL-8/CXCL8, and CCL5, which regulate immune response (66). Many of these pro-inflammatory cytokines increase the recruitment of innate immune cells and progenitor cells that are required for tissue remodeling and blood vessel development (34, 51, 67). MSC^E−selectin−GFP^ demonstrated increased upregulation in the inflammatory cytokines IL-1β and IL-6, indicating that our supercharged stem cells are of the MSC1 phenotype and have increased paracrine effects when compared with control, *in vitro*. Yet, there are other beneficial effects to the immunomodulation induced by the E-Selectin-overexpressing MSCs. We observed that TNF was downregulated in ischemic limb tissues treated with MSC^E−selectin−GFP^ while there was overlap in some pro-angiogenic genes upregulated both in the transduced MSCs and the tissues treated with these cells, the additional effects in gene expression observed only in the treated tissues (and not the cells) point the complex activation of cell-cell and paracrine secondary signaling cascades. Yet, the cells exert the tissue repair within the first 21 days, thereafter mostly being cleared. The LDI imaging quantitative data suggest that the most robust timeframe for angiogenesis occurs in the 1st week, which is likely when the MSC^E−selectin−GFP^ is most actively secreting chemokines/cytokines and modulating the ischemic tissue environment. This augmented angiogenesis is likely due to the enhanced MSCs phenotypes as evidenced by their inflammatory genetic upregulation, which potentially translates into the sustained pro-angiogenic genetic regulation within the ischemic tissues. In fact, a burst of self-limiting inflammation is essential in normal healing (68). Interestingly, although MSC^E−selectin−GFP^ produces increased levels of IL-6 *in vitro*, the treated ischemic limb tissues do not appear to have sustained inflammation and actually by day 21, downregulate TNF. Moreover, tissue levels of IL-6 in treated ischemic limbs are not increased as assessed by PCR array. Future studies are planned to perform transcriptome analysis to further elucidate the alterations seen in the MSC^E−selectin−GFP^ as other therapeutic avenues may yet be uncovered.

The fact that only a limited amount of MSCs survived in ischemic limbs 3 weeks after treatment also supports that the therapeutic effects of engrafted supercharged MSCs are achieved through early direct and paracrine mechanisms rather than permanent cell differentiation and engraftment into tissue. For CLI application, a short period of persistence of these otherwise not naturally occurring MSCs in the treated limb tissues is beneficial since it may imply less risk of long-term tumorigenesis, and thus a safe approach. Biosafety of our E-selectin overexpressing MSC therapy is also suggested by the lack of systemic biodistribution and no evidence of aberrant metabolic changes as indicated by blood tests. Additionally, no untoward side-effects were observed in any of the mice treated with MSCs during the 3-week time frame. Thus, we demonstrate that this supercharged MSC therapy has superior therapeutic efficacy and reassuring biosafety in the mouse model.

In conclusion, this study reports a novel approach at genetic manipulation of MSCs *via* a rAAV vector that induces E-selectin overexpression. These new “supercharged” MSCs grow faster and induced a number of biologic readouts that are relevant to the treatment of CLI. We have shown that our engineered rAAV vector results in efficient expression of E-selectin, which when utilized as a gene-modified cell-based treatment for hind limb ischemia, results in improved functional recovery, muscle size, angiogenesis, and tissue repair. Our study highlights this novel gene therapy approach to CLI as a potential therapeutic option to be tested in future clinical studies.

## Data Availability Statement

The raw data supporting the conclusions of this article will be made available by the authors, without undue reservation.

## Ethics Statement

The animal study was reviewed and approved by IACUC protocol 16-188.

## Author Contributions

HQ: study design, investigation, data analysis, and manuscript writing. RL-S: study design and critical review. YL, SV, YO, PP, and HS: investigation and critical review. RV-P: conceptualization and methodology. Z-JL and OV: conceptualization, methodology, validation, analysis, critical review, and editing of manuscript. All authors contributed to the article and approved the submitted version.

## Funding

This work was supported by grants from the National Institutes of Health [VITA (NHLBl-CSB-HV-2017-01-JS) and R01DK-071084, R01GM081570].

## Conflict of Interest

Z-JL and OV along with the University of Miami hold intellectual property of the AAV engineered E-selectin vector and have been licensed to Ambulero, Inc. The remaining authors declare that the research was conducted in the absence of any commercial or financial relationships that could be construed as a potential conflict of interest.

## Publisher's Note

All claims expressed in this article are solely those of the authors and do not necessarily represent those of their affiliated organizations, or those of the publisher, the editors and the reviewers. Any product that may be evaluated in this article, or claim that may be made by its manufacturer, is not guaranteed or endorsed by the publisher.
