# Supplementary material for: E-Selectin-Overexpressing Mesenchymal Stem Cell Therapy Confers Improved Reperfusion, Repair, and Regeneration in a Murine Critical Limb Ischemia Model
Source: Front Cardiovasc Med. 2022 Jan 31;8:826687. doi: 10.3389/fcvm.2021.826687 (PMC8841646; doi:10.3389/fcvm.2021.826687)
Supplement: Supplementary file 1 [file Data_Sheet_1.docx]

**Supplemental Figures/Tables.**

**Supplemental Table 1.**

| **Biochemistry Test** | **Vehicle** | **MSC^GFP^** | **MSC^E-selectin-GFP^** | **p-value** | **Reference Value** |
| --- | --- | --- | --- | --- | --- |
| White Blood Count X 10^3^/µL | 7.2 | 8.1 | 8.0 | 0.8 | 4.5-9.1 |
| Hemoglobin (g/dL) | 12.2 | 12.7 | 12.9 | 0.11 | 12.8-16.1 |
| BUN (mg/dL) | 20.0 | 25.0 | 24.0 | 0.11 | 18-29 |
| Creatinine (mg/dL) | 0.2 | 0.2 | 0.2 | 1.0 | 0.1-0.4 |
| Phosphorus (mg/dL) | 10.0 | 10.0 | 8.9 | 0.33 | 5.4-9.3 |
| AST (U/L) | 165 | 123 | 82 | 0.06 | 50-270 |
| ALT (U/L) | 28 | 36 | 47 | 0.39 | 29-77 |
| Total Bilirubin | 0.7 | 0.4 | 0.3 | 0.25 | 0.1-0.9 |

Blood counts, electrolyte, and liver and kidney function blood tests were performed via intracardiac draw before animal sacrifice, at post-operative day 21. There was no difference found between mice treated with MSC^E-selectin-GFP^ (n=8), MSC^GFP^ (n=4), or vehicle (n=3) via one-way ANOVA statistical analysis.

**Supplemental Table 2.**

| **Symbol** | **Description** | **Mean of Ct** | **p-value** |
| --- | --- | --- | --- |
| HGF | Hepatocyte growth factor | 0.73 | ns |
| PDGF-a | Platelet derived growth factor, alpha | 0.24 | ns |
| VEGF-a | Vascular endothelial growth factor A | 0.33 | ns |
| VEGF-b | Vascular endothelial growth factor B | 0.34 | ns |
| VEGF-c | Vascular endothelial growth factor C | 0.35 | ns |

Levels of several angiogenic genes, including VEGF, HGF and PDGF, were unchanged in the cell culture of MSC^E-selectin-GFP^ compared to MSC^GFP^ in vitro according to RT^2^-PCR-*Array* by mean of Ct via Two-tailed Student’s T tests. ns: not significant. Cutting-off value is ≥ 1.

**Supplemental Figure 1.**


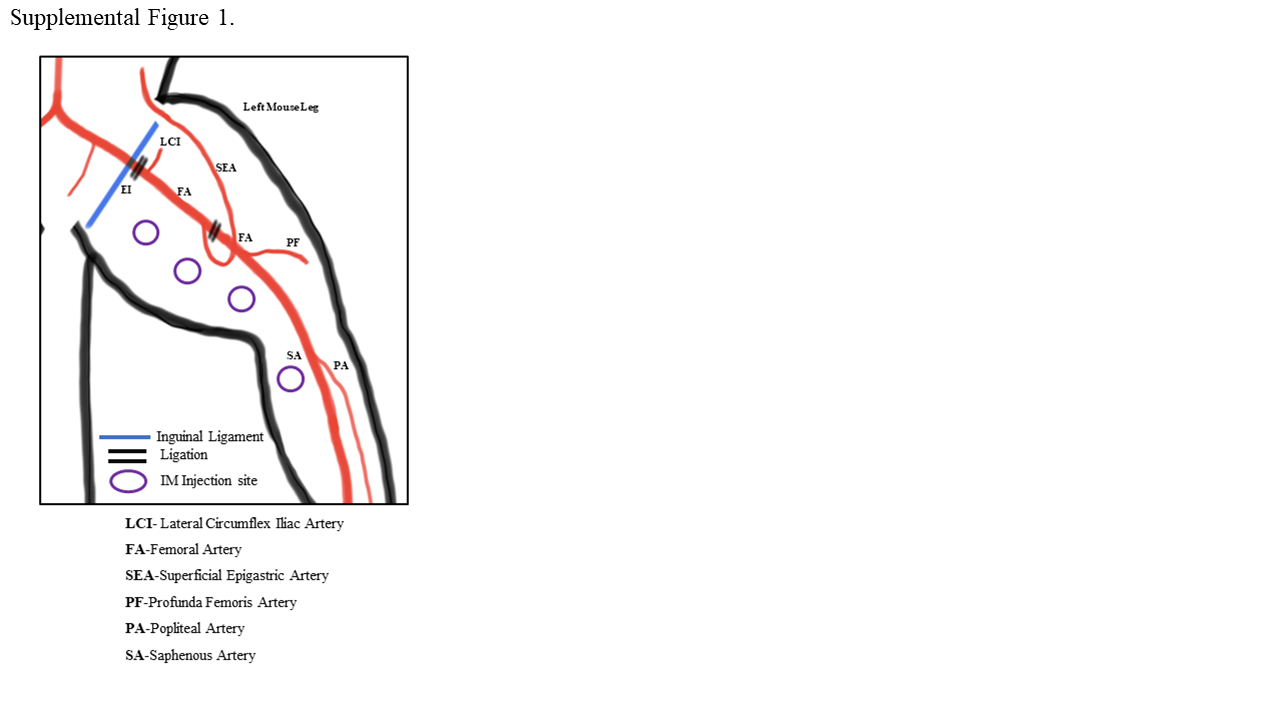


**Supplemental Figure 1.** Peripheral vascular anatomy in mice. Femoral Artery ligation (FAL) performed via proximal and distal ligation, which can be seen as = on figure, they’re located below the inguinal ligament and immediately above the sapheno-popliteal bifurcation, respectively. Intramuscular injection (IM) sites are located in 4 areas in the left (ligated) medial thigh and calf muscles.
